# Treatment patterns and survival among older adults in the United States with advanced soft-tissue sarcomas

**DOI:** 10.1186/s13569-018-0094-x

**Published:** 2018-05-03

**Authors:** Rohan C. Parikh, Maria Lorenzo, Lisa M. Hess, Sean D. Candrilli, Steven Nicol, James A. Kaye

**Affiliations:** 1 RTI Health Solutions, 200 Park Offices Drive, Research Triangle Park, NC 27560 USA; 2 Eli Lilly and Company Limited, Lilly Research Centre, Erl Wood Manor, Sunninghill Road, Windlesham, Surrey, GU20 6PH UK; 3 Eli Lilly and Company, Lilly Corporate Center, Indianapolis, IN 46285 USA; 4 RTI Health Solutions, 307 Waverley Oaks Road, Suite 101, Waltham, MA 02452 USA

**Keywords:** Soft-tissue sarcoma, Chemotherapy, Observational study

## Abstract

**Background:**

To describe patient and tumor characteristics, treatments, and survival among older adults in the United States with advanced soft-tissue sarcoma (STS), across and by categories of specifically defined histologic subtypes.

**Methods:**

We conducted a retrospective cohort analysis using the SEER. The study population comprised patients ≥ 65 years old with advanced STS (excluding osteosarcoma, Kaposi sarcoma, and gastrointestinal stromal tumors) diagnosed from January 1, 2001 to December 31, 2011.

**Results:**

Of 4274 study patients, 2103 (49.2%) were male. Mean age was 77.8 years, and 1539 (36.0%) had distant disease at initial diagnosis. The most common histologic categories were leiomyosarcoma (922[21.6%]), undifferentiated pleomorphic sarcoma (652[15.3%]), and liposarcoma (554[13.0%]). Overall, 1227 (28.7%) patients received first-line systemic therapy. Among these patients, 325 (26.5%) received docetaxel plus gemcitabine and 231 (18.8%) received doxorubicin alone. Only 476 patients received second-line therapy (11.1%), most commonly doxorubicin alone (n = 101). Median overall survival (95% confidence interval) from advanced STS diagnosis was 8.9 (8.3, 9.7) months.

**Conclusions:**

Although previous studies of younger populations reported anthracycline-based therapy predominated in first line, our study of older advanced STS patients found that docetaxel plus gemcitabine was most commonly used. Despite variation by histologic category, prognosis remains poor for older adult patients with advanced STS.

**Electronic supplementary material:**

The online version of this article (10.1186/s13569-018-0094-x) contains supplementary material, which is available to authorized users.

## Background

Soft-tissue sarcoma (STS) refers to a rare and heterogeneous group of malignant tumors comprising more than 50 histologic subtypes derived from connective tissues and other cells of mesenchymal origin. Soft-tissue sarcoma accounts for approximately 1% of all incident malignancies [1] and an estimated 13,040 new cases will be diagnosed in the United States (US) in 2018, with 7370 dying from the disease [2]. In 2014, the age-adjusted incidence rate, for STS for patients 65 years of age and older was 11.3 per 100,000, compared to 2.3 per 100,000 for patients younger than 65 years of age [3].

Among all patients diagnosed with STS, the 5-year overall survival is approximately 50% [4]. Tumor histology, stage, and primary site are prognostic factors [4, 5]; 5-year overall survival is 83% in patients with localized disease and 16% in patients with distant metastases at initial diagnosis [6]. Eventually, either at initial diagnosis or after recurrence from a more limited extent of disease, 40–50% of patients with STS will have metastatic or unresectable locally-advanced disease (collectively referred to as “advanced STS”) [7].

Treatment options for patients with advanced STS have historically been limited; cytotoxic chemotherapy has been the mainstay of therapy for decades. Active drugs include doxorubicin, ifosfamide, gemcitabine, taxanes, and several others; these may be administered either as single agents or in combination regimens [4, 8]. The intent of these treatments is palliative for the majority of patients [4, 8, 9]. Until recently, clinical trials—including those demonstrating superiority of combination chemotherapy over monotherapy and those leading to the regulatory approval of newer agents such as olaratumab, trabectedin, and pazopanib—have found improvements in response rates and progression-free survival [4, 10–14]. However, olaratumab is the only newer agent to date that has also demonstrated a statistically significant improvement in overall survival [11].

Few studies have examined utilization of systemic treatments for STS in the general population of the US, especially in older patients with advanced disease. Furthermore, there is little published information on variation in outcomes according to tumor histology, especially among older adults. Thus, the objectives of this study were to describe real-world, population-based treatment patterns and survival among older adult patients with advanced STS, overall and by groupings of specific histologic subtypes (hereafter “histologic categories”).

## Methods

### Data source

The Surveillance, Epidemiology, and End Results (SEER), which contains administrative claims data for Medicare enrollees, was used for the study. The SEER cancer registry comprises nine state population-based and seven metropolitan or regional population-based registries that routinely collect information on 98% of newly diagnosed (incident) cancer cases, in persons residing in SEER areas [15]. SEER areas have been shown to be nationally representative [16] and capture approximately one-quarter of the total US population [15].

Along with detailed information on incident cancer, the database provides comprehensive longitudinal health care utilization data from Medicare. The Medicare claims database provides information on all services covered under Medicare Part A benefits, including inpatient, skilled nursing facility, home health care, and hospice care. In addition, the database provides information on approximately 95% of services covered under Medicare Part B, including physician visits, outpatient care, durable medical equipment, and home health care. This study was deemed exempt from Institutional Review Board review in accordance with the US Code of Federal Regulations [45CFR46.101(b)] as these data do not contain any variables that could identify an individual subject either directly or indirectly.

### Study population

Patients with a new diagnosis of STS between January 1, 2001 and December 31, 2011 were eligible for this study. Medicare claims data were available through 2013. Patients with STS (except osteosarcoma, Kaposi sarcoma, and gastrointestinal stromal tumors) were identified in the SEER database using International Classification of Diseases for Oncology, 3rd Edition (ICD-O-3), morphology codes as listed in Appendix 1: Table 6 [17]. In order to reduce the number of strata analyzed specific histologic subtypes (i.e., individual ICD-O-3 codes) were grouped into broader categories for analysis based on the 2013 World Health Organization’s (WHO) Classification of Tumours of Soft Tissue and Bone [18]: (1) leiomyosarcoma (smooth muscle tumors), (2) undifferentiated pleomorphic sarcoma (UPS; previously known as malignant fibrous histiocytoma), (3) liposarcoma (adipocytic tumors), (4) vascular sarcomas, (5) fibroblastic/myofibroblastic sarcoma, (6) nerve sheath sarcoma, (7) rhabdomyosarcoma (skeletal muscle tumors), (8) synovial sarcoma, and (9) others or not otherwise specified (NOS). The term “histologic category” was used in this study to distinguish these categories from the individual “histologic subtypes” they contain.

Advanced disease was defined as metastatic disease or as the presence of nodal metastases and no surgery (assuming patients who did not receive surgery were not suitable for surgical resection). Metastatic disease and nodal metastases were identified using the American Joint Commission on Cancer’s Cancer Staging Manual [19] or the National Cancer Institute’s (NCI’s) SEER Summary Staging Manual [20]. Patients were also considered to have advanced STS if they had an initial diagnosis of localized disease with later progression to advanced STS as identified by either a medical claim with an International Classification of Diseases, 9th Revision, Clinical Modification (ICD-9-CM) diagnosis code for a secondary neoplasm (ICD-9-CM 196.0-198.89) or initiation of systemic therapy more than 6 months after surgery. The 6-month lag period was chosen to avoid interpreting adjuvant therapy as treatment for advanced disease.

Additionally, eligible patients were 65 years or older at the time of advanced STS diagnosis and were enrolled in both Medicare parts A and B without any health maintenance organization (HMO) enrollment from 6 months before initial STS diagnosis to death or end of study. Patients with HMO coverage (provided outside of the Medicare system) were excluded because claims data for these patients would not be complete, as the linked data for this study were limited to Medicare. Of the patients initially diagnosed with localized or regional disease according to SEER data, only those with evidence of advanced STS in Medicare claims that occurred at a date later than the initial STS diagnosis were included in the study (i.e., patients were excluded if there were contradictory initial diagnosis codes [i.e., localized or regional STS diagnosis and secondary neoplasm claims] on the same date, or if patients had no surgery after initial STS diagnosis and no secondary neoplasm claim). Study index date for each patient (start of follow-up) was defined as the date of the first observed evidence of advanced STS.

### Demographic and clinical characteristics

Demographic characteristics including age, sex, race, SEER registry location, and urban or rural residency were tabulated. Clinical characteristics that were assessed at initial STS diagnosis included cancer stage, tumor location, and histologic subtype (with subsequent categorization described previously). We also computed a modified Charlson Comorbidity Index (CCI) score to obtain a measure of each patient’s overall comorbidity burden at the time of the index diagnosis [21–23]. CCI scores were calculated for each patient based on evidence of the relevant diagnoses from 6-months prior to advanced STS diagnosis to advanced STS diagnosis (i.e., during 6-month pre-index date period). ICD-9-CM diagnosis codes for cancer were excluded from the CCI calculation for this study so as to not overestimate the baseline comorbidity burden, as all patients in this study had cancer.

### Treatment patterns

Treatments were identified using evidence of relevant Healthcare Common Procedure Coding System (HCPCS) codes, ICD-9-CM procedure codes, and certain ICD-9-CM diagnostic codes and administrative revenue codes. In addition to HCPCS and ICD-9-CM codes, Medicare Part D prescription data were used to identify chemotherapy use for a subset of the cohort with this data (2007–2012). Among patients who received systemic treatment, up to five lines of treatment were identified. Specific therapies were examined based upon options included in the National Comprehensive Cancer Network (NCCN) Guidelines [24]. First-line treatment was examined among all patients, with at least one claim for an HCPCS-identifiable chemotherapeutic, biologic, or targeted therapy agent after their index date. The first observed date of treatment after the index date signaled the start of first-line treatment. First-line treatment was defined as the combination of all agents observed within 21 days after (and inclusive of) treatment initiation. After the end of this 21-day period, the use of any new agent signaled the start of the next line of treatment. Discontinuation of one or more of the agents without the addition of another agent was not considered a new line of treatment. In addition, patients with at least 6 months between apparent cessation of their previous line of treatment and reuptake of an identical regimen were defined as having initiated a next line of treatment. End of a line of treatment was defined as: (1) a 6-month gap in treatment regimen, (2) interruption by next line of treatment, or (3) treatment continuation until the end of study or death. Cancer-directed therapy was defined as receipt of any of broad treatment categories of surgery, radiation, chemotherapy, biologic therapy, or targeted therapy, while supportive care was defined as no receipt of treatment from any of the broad cancer-directed therapies.

### Statistical analyses

Descriptive statistics [i.e., means, medians, ranges, and standard deviations (SDs) of continuous variables and frequency counts and percentages for categorical variables] were computed. Overall survival from advanced STS diagnosis was measured using the death information available from SEER and Medicare data, and survival time end points were analyzed using the Kaplan–Meier estimates with median survival time and 95% confidence intervals (CIs) reported in months. Patients alive at the end of study period were censored for this analysis. Treatment patterns and survival were assessed in the overall advanced STS population and by histologic category. Additionally, survival was estimated separately for patients who received cancer-directed treatment and those who received supportive care only. All analyses were conducted using SAS Version 9.4 (Cary, NC: SAS Institute, Inc.; 2011). The SEER-Medicare data use agreement required that no cell sizes less than 11 be reported for any demographic or other characteristic, or combination of characteristics; thus, if sufficient sample size was not available, data were not reported and indicated with “–”.

## Results

A total of 4274 patients with advanced STS met the inclusion criteria for this study (Table 1). Patients were on average 77.8 years of age (SD, 7.3; range 65–104 years) at the time of advanced STS diagnosis and 49.2% were male (Table 2). The mean (SD) CCI score was 2.8 (2.33), and 69.2, 18.3, and 6.5% of patients had a history of hypertension, congestive heart failure, and myocardial infarction, respectively.

**Table 1:** Study population selection process

	Number of patients
Initial sample included in the SEER database	142,689
Initial diagnosis date on or after January 1, 2001	135,608
Patients whose first diagnosis recorded in the SEER database was STS	21,167
Patients with evidence (i.e., claim in Medicare data or diagnosis in SEER database) of metastatic disease	8537
Initially diagnosed at metastatic stage^a^	3391
Initially diagnosed at non-metastatic stage^a^	5146
Patients 65 years or older at time of metastatic STS diagnosis	6712
Patients who were alive at initial diagnosis of STS (i.e., reporting source other than autopsy or death certificate)	6705
Patients who were not enrolled in an HMO for at least 6 months prior to initial diagnosis of STS to end of follow-up period	5166
Patients who have continuous enrollment in Medicare Part A and B (non-HMO) for at least 6 months prior to initial diagnosis of STS until end of follow-up period	4353
Patients who are not lost to follow-up prior to the assigned advanced STS diagnosis date^b^	4324
Patients with a date of advanced STS diagnosis different than initial STS diagnosis (patients excluded if no surgery after initial STS diagnosis and no secondary neoplasm claim)	4298
Patients with a date of advanced STS diagnosis different than initial STS diagnosis (patients excluded if initial STS diagnosis and secondary neoplasm claim on the same date)	4274
*Final study population*	*4274*

*HMO* health maintenance organization, *SEER* Survey, Epidemiology, and End Results, *STS* soft-tissue sarcoma

^a^Only for descriptive purposes and no exclusion was made based on this criterion

^b^Day of diagnosis, which is reported as month and years, was assigned as 15th of each month and hence some patients who either die or are lost to follow-up before the 15th of the month have a negative length of follow-up

**Table 2:** Patient and tumor characteristics, overall and by histologic categories

	All patients	Leiomyo-sarcomas	Undifferentiated pleomorphic sarcoma	Liposarcomas	Vascular sarcomas	Fibroblastic/myofibroblastic sarcomas	Nerve sheath sarcomas	Rhabdomyo-sarcomas	Synovial sarcomas	Others/NOS
N	4274	922	652	554	357	227	106	98	49	1309
Sex (n, %)										
Male	2103 (49.2)	307 (33.3)	390 (59.8)	329 (59.4)	195 (54.6)	122 (53.7)	64 (60.4)	43 (43.9)	28 (57.1)	625 (47.8)
Female	2171 (50.8)	615 (66.7)	262 (40.2)	225 (40.6)	162 (45.4)	105 (46.3)	42 (39.6)	55 (56.1)	21 (42.9)	684 (52.2)
Race										
White	3729 (87.3)	783 (84.9)	576 (88.3)	499 (90.1)	309 (86.6)	203 (89.4)	96 (90.6)	82 (83.7)	43 (87.8)	1138 (86.9)
Black	337 (7.9)	106 (11.5)	46 (7.1)	23 (4.2)	21 (5.9)	–	–	–	–	106 (8.1)
Other	197 (4.6)	31 (3.4)	26 (4.0)	32 (5.8)	25 (7.0)	–	–	–	–	63 (4.8)
Age at *advanced* diagnosis										
Mean (SD)	77.8 (7.3)	76.8 (7.2)	79.4 (7.2)	77.5 (7.1)	78.2 (7.5)	77.8 (7.1)	77.7 (7.6)	76.8 (7.3)	74.1 (6.5)	78.0 (7.4)
Median	77.4	76.1	79.7	76.6	77.9	77.8	77.3	76.6	73.5	77.5
Min, Max	65.0, 104.1	65.0, 102.9	65.1, 99.3	65.1, 96.8	65.6, 101.8	65.7, 97.4	65.5, 95.9	65.3, 95.3	65.7, 91.0	65.1, 104.1
Pre-index date Charlson Comorbidity Index (CCI) score^a^										
Mean (SD)	2.8 (2.3)	2.5 (2.2)	2.8 (2.4)	2.8 (2.3)	2.9 (2.7)	2.8 (2.2)	2.8 (2.3)	2.6 (2.1)	3.0 (2.3)	2.8 (2.4)
Median	2.0	2.0	2.0	2.0	2.0	2.0	2.0	2.0	2.0	2.0
Min, Max	0.0, 15.0	0.0, 14.0	0.0, 15.0	0.0, 12.0	0.0, 12.0	0.0, 11.0	0.0, 10.0	0.0, 9.0	0.0, 8.0	0.0, 15.0
Stage of disease at initial diagnosis of STS (n, %)										
Localized	1731 (40.5)	345 (37.4)	379 (58.1)	260 (46.9)	150 (42.0)	121 (53.3)	36 (34.0)	25 (25.5)	18 (36.7)	397 (30.3)
Regional—direct extension only	938 (22.0)	200 (21.7)	135 (20.7)	177 (32.0)	62 (17.4)	59 (26.0)	32 (30.2)	14 (14.3)	11 (22.5)	248 (19.0)
Regional—lymph nodes involved only	29 (0.7)	–	–	–	–	–	–	–	0 (0.0)	–
Regional—direct extension and lymph nodes	37 (0.9)	–	–	–	–	–	–	–	0 (0.0)	–
Distant	1539 (36.0)	363 (39.4)	133 (20.4)	114 (20.6)	138 (38.7)	45 (19.8)	36 (34.0)	53 (54.1)	20 (40.8)	637 (48.7)
Grade of cancer at time of initial diagnosis of STS (n, %)										
Well or moderately differentiated (grade 1 or 2)	646 (15.1)	152 (16.5)	36 (5.5)	239 (43.1)	40 (11.2)	50 (22.0)	–	–	–	116 (8.9)
Poorly differentiated (grade 3)	908 (21.2)	192 (20.8)	151 (23.2)	101 (18.2)	81 (22.7)	35 (15.4)	–	–	16 (32.7)	294 (22.5)
Undifferentiated (grade 4)	1310 (30.7)	250 (27.1)	228 (35.0)	129 (23.3)	61 (17.1)	63 (27.8)	28 (26.4)	34 (34.7)	–	510 (39.0)
Unknown	1410 (33.0)	328 (35.6)	237 (36.4)	85 (15.3)	175 (49.0)	79 (34.8)	54 (50.9)	39 (39.8)	24 (49.0)	389 (29.7)
Total follow-up time (years)^b^										
Mean (SD)	1.7 (2.4)	1.8 (2.2)	2.0 (2.8)	2.6 (2.8)	1.1 (1.7)	2.5 (2.7)	1.4 (2.1)	0.8 (1.5)	1.4 (1.8)	1.3 (2.0)
Median	0.7	1.0	0.8	1.5	0.5	1.6	0.5	0.3	0.6	0.5
Min, Max	0.0, 12.8	0.0, 12.2	0.0, 12.3	0.0, 12.3	0.0, 12.5	0.0, 12.5	0.0, 9.9	0.0, 9.8	0.0, 8.8	0.0, 12.8

*ICD-9-CM* International Classification of Diseases, 9th Revision, Clinical Modification, *Max* maximum, *Min* minimum, *NOS* not otherwise specified, *SD* standard deviation, *STS* soft-tissue sarcoma

^a^Because the objective of the CCI score was to evaluate underlying comorbidity burden independent of STS, ICD-9-CM diagnosis codes for cancer were excluded from the CCI calculation for this study

^b^Follow-up time calculated as the number of months between the study index date (first diagnosis of advanced STS) and the earliest of death, loss of eligibility, or end of the Medicare database (December 31, 2013)

At initial diagnosis, 36.0% of patients had metastatic (distant) disease; the remaining majority (64.0%) were diagnosed at earlier stages of disease and had claims based indicators of progression to advanced STS (Table 2) with a mean (SD) interval of 16.6 (23.0) months from initial diagnosis. Nodal disease (without any distant metastases) was observed only among 1.6% of patients at initial diagnosis. The most common histologic category was leiomyosarcoma (n = 922; 21.6%), followed by UPS (n = 652; 15.3%), liposarcoma (n = 554; 13.0%), vascular sarcoma (n = 357; 8.4%), fibroblastic/myofibroblastic sarcoma (n = 227; 5.3%), nerve sheath sarcoma (n = 106; 2.5%), rhabdomyosarcoma (n = 98; 2.3%), and synovial sarcoma (n = 49; 1.1%); the remaining 30.6% (n = 1309) had other or NOS histologic categories. A few variations in patient and tumor characteristics by tumor histology were evident: 66.7% of patients with leiomyosarcoma were female; patients with synovial sarcoma had a mean age of 74.1 years, and 54.1, 40.8, 48.7% of patients with rhabdomyosarcoma, synovial sarcoma, and other/NOS histology, respectively, had distant stage of disease at initial STS diagnosis (Table 2). Overall, the most common specified anatomic tumor site was lower limb (21.9%) (Appendix 2: Table 7), and the most common known tumor grade was undifferentiated (30.7%) (Table 2). The average observed follow-up time from advanced STS diagnosis was 1.7 years (SD, 2.36) (Table 2).

Cancer-directed treatment was received by 62.1% of patients. Radiation was received by 40.0% of patients and surgery was received by 10.3% of patients. The mean (SD) age of patients who received cancer-directed treatment was 76.5 (6.9) years and the mean (SD) CCI score was 2.5 (2.1), with hypertension, diabetes (without complications), chronic pulmonary disease, peripheral vascular disease, and congestive heart failure observed among 68.9, 28.4, 24.4, 18.1, and 14.6% of patients, respectively (Additional file 1: Table S1). Chemotherapy, biologic therapy, and targeted therapy were received by 27.5, 1.9, and 1.3% of patients, respectively. Among the 28.7% of all patients with advanced STS who received chemotherapy, biologic therapy, or targeted therapy (n = 1227), the mean duration of first-line therapy was 4.1 months (SD, 4.1) (Table 3). Second-line therapy was received by 11.1% (n = 476) of all patients; the mean duration of second-line treatment was 4.6 months (SD, 5.3). Among the 4.4% of all patients with advanced STS who received at least third-line therapy (n = 189), the mean duration of third-line therapy was 4 months (SD, 3.4). The proportion of patients receiving up to three lines of treatment and duration of each line of treatment by histologic category is shown in Table 3. First-line chemotherapy was most commonly received by patients with synovial sarcoma (42.9%), leiomyosarcoma (37.0%), and vascular sarcoma (36.4%).

**Table 3:** Treatment line progression, overall and by histologic categories

	Histologic categories									
	All patients	Leiomyo-sarcomas	Undifferentiated pleomorphic Sarcoma	Liposarcomas	Vascular sarcomas	Fibroblastic/myofibroblastic sarcomas	Nerve sheath sarcomas	Rhabdomyo-sarcomas	Synovial sarcomas	Others/NOS
	n (%)	n (%)	n (%)	n (%)	n (%)	n (%)	n (%)	n (%)	n (%)	n (%)
All patients	4274	922	652	554	357	227	106	98	49	1309
Receiving first-line systemic^a^ treatment	1227 (28.7)	341 (37.0)	158 (24.2)	140 (25.3)	130 (36.4)	58 (25.6)	24 (22.6)	28 (28.6)	21 (42.9)	327 (25.0)
Duration, mean (SD)	4.1 (4.1)	4.1 (4.2)	4.4 (5.7)	4.3 (4.7)	4.4 (3.4)	4.2 (3.8)	3.3 (2.5)	4.5 (2.9)	3.9 (3.3)	3.8 (3.2)
Median	3.0	3.1	2.6	3.0	3.5	3.6	3.1	3.7	3.0	2.8
Receiving second-line systemic^a^ treatment	476 (11.1)	148 (16.1)	64 (9.8)	45 (8.1)	60 (16.8)	19 (8.4)	–	15 (15.3)	–	109 (8.3)
Duration, mean (SD)	4.6 (5.3)	4.5 (4.0)	5.1 (8.6)	5.5 (5.4)	4.6 (5.2)	4.7 (3.6)	–	3.2 (2.2)	–	4.3 (4.7)
Median	3.0	3.3	2.9	4.0	3.4	4.8	–	2.6	–	2.9
Receiving third-line systemic^a^ treatment	189 (4.4)	66 (7.2)	24 (3.7)	15 (2.7)	21 (5.9)	–	–	–	–	46 (3.5)
Duration, mean (SD)	4.0 (3.4)	4.6 (3.6)	3.3 (3.8)	2.8 (2.2)	5.1 (3.6)	–	–	–	–	3.4 (2.8)
Median	3.0	3.7	2.3	2.1	5.1	–	–	–	–	2.6

Oral chemotherapy was not covered by Medicare until 2007

*SD* standard deviation

^a^Systemic therapy includes chemotherapy, biologic therapy, or targeted therapy

Overall, the most common regimen during first-line therapy was docetaxel plus gemcitabine (26.5%), followed by doxorubicin monotherapy (18.8%), gemcitabine monotherapy (9.1%), paclitaxel monotherapy (5.5%), and bevacizumab monotherapy (3.3%). Docetaxel plus gemcitabine was the most common first-line therapy for patients with all histologic categories except liposarcoma (for whom it was doxorubicin monotherapy [24.3%]) and vascular sarcoma (for whom it was paclitaxel monotherapy [40.0%]) (Table 4). During first-line therapy, doxorubicin plus ifosfamide was received by 3.0% and ifosfamide monotherapy was received by 2.7% of patients. Among all patients who received doxorubicin alone or a doxorubicin-based first-line therapy (n = 371), 29.7% received liposomal doxorubicin, and 5.9% received the cardioprotective agent dexrazoxane.

**Table 4:** Top 5 most frequent regimens during first-, second-, and third-line therapy

First-line therapy	n (%)	Second-line therapy	n (%)	Third-line therapy	n (%)
All patients (N = 1227)		All patients (N = 476)		All patients (N = 189)	
Docetaxel–gemcitabine	325 (26.5)	Doxorubicin	101 (21.2)	Doxorubicin	28 (14.8)
Doxorubicin	231 (18.8)	Docetaxel–gemcitabine	83 (17.4)	Docetaxel–gemcitabine	21 (11.1)
Gemcitabine	112 (9.1)	Gemcitabine	41 (8.6)	Gemcitabine	21 (11.1)
Paclitaxel	68 (5.5)	Paclitaxel	21 (4.4)	Ifosfamide	15 (7.9)
Bevacizumab	41 (3.3)	Ifosfamide	20 (4.2)	Dacarbazine	–

Leiomyosarcomas (N = 341)		Leiomyosarcomas (N = 148)		Leiomyosarcomas (N = 66)	
Docetaxel–gemcitabine	136 (39.9)	Doxorubicin	41 (27.7)	Doxorubicin	12 (18.2)
Doxorubicin	64 (18.8)	Docetaxel–gemcitabine	35 (23.7)	Docetaxel–gemcitabine	–
Gemcitabine	43 (12.6)	Gemcitabine	–	Gemcitabine	–
Carboplatin-paclitaxel	–	Docetaxel	–	Carboplatin-paclitaxel	–
Dacarbazine-doxorubicin	–	Dacarbazine	–	Ifosfamide	–
Doxorubicin–ifosfamide	–				
Temozolomide	–				

Undifferentiated pleomorphic sarcoma (N = 158)		Undifferentiated pleomorphic sarcoma (N = 64)		Undifferentiated pleomorphic sarcoma (N = 24)	
Docetaxel–gemcitabine	36 (22.8)	Docetaxel–gemcitabine	1 3 (20.3)	Doxorubicin	–
Doxorubicin	32 (20.3)	Doxorubicin	–	Docetaxel–gemcitabine	–
Gemcitabine	13 (8.2)	Ifosfamide	–	Dacarbazine	–
Ifosfamide	11 (7.0)	Gemcitabine	–	Ifosfamide	–
Doxorubicin–ifosfamide	–	Paclitaxel	–		
		Temozolomide	–		

Liposarcomas (N = 140)		Liposarcomas (N = 45)		Liposarcomas (N = 15)	
Doxorubicin	34 (24.3)	Docetaxel–gemcitabine	11 (24.4)	Ifosfamide	–
Docetaxel–gemcitabine	31 (22.1)	Doxorubicin	–	Gemcitabine	–
Bevacizumab	13 (9.3)	Gemcitabine	–		
Gemcitabine	12 (8.6)	Docetaxel-doxorubicin-gemcitabine	–		
Doxorubicin–ifosfamide	–	Ifosfamide	–		
Ifosfamide	–				

Vascular sarcomas (N = 130)		Vascular sarcomas (N = 60)		Vascular sarcomas (N = 21)	
Paclitaxel	52 (40.0)	Doxorubicin	18 (30.0)	Doxorubicin	–
Doxorubicin	22 (16.9)	Paclitaxel	–	Doxorubicin–ifosfamide	–
Docetaxel–gemcitabine	–	Docetaxel–gemcitabine	–	Docetaxel	–
Doxorubicin–ifosfamide	–	Gemcitabine	–	Docetaxel–gemcitabine	–
Docetaxel	–	Docetaxel	–	Paclitaxel	–

Fibroblastic/Myofibroblastic sarcomas (N = 58)		Fibroblastic/Myofibroblastic sarcomas (N = 19)		Fibroblastic/Myofibroblastic sarcomas (N = –)	
Docetaxel–gemcitabine	16 (27.6)	Bevacizumab	–	Docetaxel–gemcitabine	–
Doxorubicin	–	Docetaxel–gemcitabine	–	Gemcitabine	–
Bevacizumab	–	Bevacizumab-temozolomide	–		
Gemcitabine	–	Gemcitabine	–		
Cisplatin	–	Temozolomide	–		
Doxorubicin–ifosfamide	–				

Nerve sheath sarcomas (N = 24)		Nerve sheath sarcomas (N = –)		Nerve sheath sarcomas (N = –)	
Doxorubicin	–	Doxorubicin	–		
Docetaxel–gemcitabine	–				
Gemcitabine	–				
Doxorubicin–ifosfamide	–				
Ifosfamide	–				

Rhabdomyosarcomas (N = 28)		Rhabdomyosarcomas (N = 15)		Rhabdomyosarcomas (N = –)	
Docetaxel–gemcitabine	–	Doxorubicin	–		
Carboplatin-paclitaxel	–	Cyclophosphamide-doxorubicin-vincristine	–		
Cyclophosphamide-dactinomycin-vincristine	–				
Cyclophosphamide-doxorubicin	–				
Cyclophosphamide-doxorubicin-vincristine	–				
Doxorubicin	–				

Synovial sarcomas (N = 21)		Synovial sarcomas (N = –)		Synovial sarcomas (N = –)	
Docetaxel–gemcitabine	–	Dacarbazine	–		
Doxorubicin	–	Doxorubicin	–		
Temozolomide	–	Ifosfamide	–		

Others/NOS (N = 327)		Others/NOS (N = 109)		Others/NOS (N = 46)	
Docetaxel–gemcitabine	81 (24.8)	Docetaxel–gemcitabine	14 (12.8)	Gemcitabine	–
Doxorubicin	61 (18.7)	Doxorubicin	14 (12.8)	Ifosfamide	–
Gemcitabine	30 (9.2)	Gemcitabine	12 (11.0)	Dacarbazine	–
Carboplatin-paclitaxel	16 (4.9)	Carboplatin-paclitaxel	–	Docetaxel–gemcitabine	–
Paclitaxel	12 (3.7)	Docetaxel	–	Doxorubicin	–
		Doxorubicin–ifosfamide	–		
		Ifosfamide	–		

Only showing treatment regimens received by more than 1 patient

*NOS* not otherwise specified

Among patients who received second-line (n = 476) and third-line (n = 189) therapy, doxorubicin monotherapy (second line, 21.2%; third line, 14.8%), docetaxel plus gemcitabine (second line, 17.4%; third line, 11.1%), and gemcitabine monotherapy (second line, 8.6%; third line, 11.1%) were most common (Table 4). Among the 476 patients who received second-line therapy, the most common treatment sequence was first-line docetaxel plus gemcitabine followed by second-line doxorubicin monotherapy (n = 56, 11.8%). Among the 189 patients who received third-line therapy, the most common sequence was first-line doxorubicin followed by second-line gemcitabine and then third-line docetaxel plus gemcitabine (n ≤ 11). Among patients who received second- or third-line therapy, ≤ 11 received dexrazoxane.

Among patients who received supportive care only (n = 1618, 37.86%), the mean (SD) age was 80.0 (7.5) years (Additional file 1: Table S1). Hypertension (69.6%), diabetes without complications (28.7%), chronic pulmonary disease (28.1%), congestive heart failure (24.5%), and peripheral vascular disease (22.6%) were the most common baseline comorbidities and the mean (SD) CCI score was 3.1 (2.6).

**Fig. 1:**
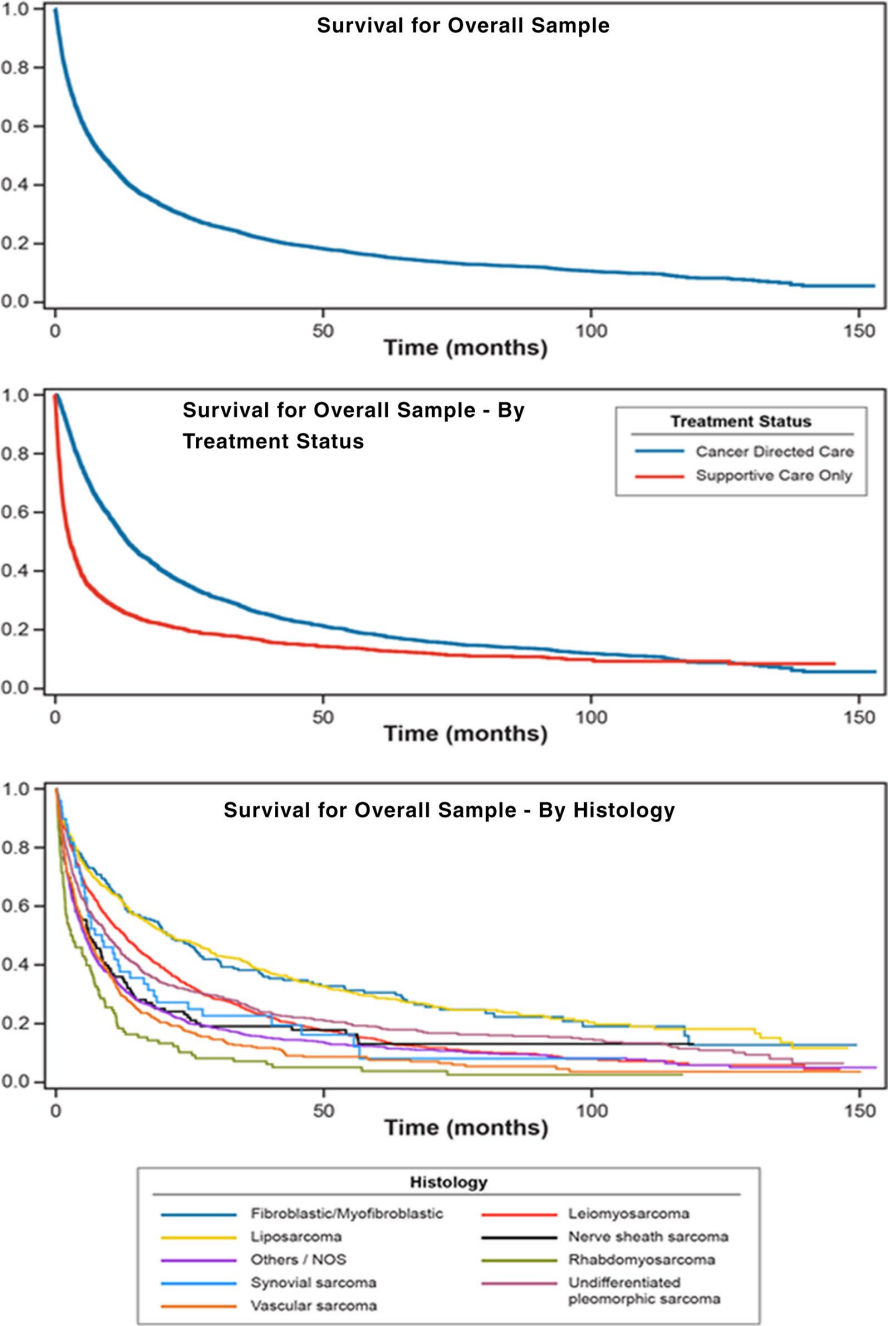
Survival estimates from diagnosis of advanced soft-tissue sarcoma

The majority of patients (n = 3565; 83.4%) died during study follow-up, with a median survival (95% CI) of 8.9 months (8.3, 9.7) from the time of advanced STS diagnosis. Median survival (95% CI) from advanced STS diagnosis among patients who received cancer-directed therapy was 13.6 months (12.9, 14.6), and among those who received supportive care it was 2.8 months (2.6, 3.4) (Table 5 and Fig. 1). Survival estimates for each histologic category is shown in Table 5. Estimated survival from advanced STS diagnosis varied by histologic category with median survival ranging from 21.4 months (15.6, 26.8) for patients with fibroblastic/myofibroblastic sarcoma to 3.0 months (1.8, 5.7) for patients with rhabdomyosarcoma.

**Table 5:** Overall survival by treatment status and histologic categories

Overall	Survival from initial diagnosis of advanced STS					
	Total	Died	Censored	Median length of survival (in months)	95% CI	
All advanced STS patients	4274	3565	709	8.9	8.3	9.7
Treatment status						
Received cancer-directed treatment	2656	2185	471	13.6	12.9	14.6
Received supportive care only	1618	1380	238	2.8	2.6	3.4
Histologic categories						
Fibroblastic/myofibroblastic sarcomas	227	160	67	21.4	15.6	26.8
Leiomyosarcomas	922	781	141	12.9	10.9	14.6
Liposarcomas	554	395	159	21.1	17.4	27.1
Nerve sheath sarcomas	106	88	18	6.2	3.6	9.3
Rhabdomyosarcomas	98	–	–	3.0	1.8	5.7
Synovial sarcomas	49	–	–	8.6	5.3	13.8
Undifferentiated pleomorphic sarcomas	652	539	113	9.6	8.2	11.4
Vascular sarcomas	357	327	30	6.0	4.8	7.3
Others/NOS	1309	1139	170	5.4	4.8	6.1

*CI* confidence interval, *NOS* not otherwise specified, *STS* soft-tissue sarcoma

## Discussion

This study assessed recent real-world treatment patterns and estimated survival of a population-based cohort of older adults diagnosed with advanced STS in the US. More than 60% of patients were initially diagnosed at an earlier stage of disease and progressed to advanced STS with mean time to progression of 16.6 months. Consistent with previous studies [25–27], leiomyosarcoma, UPS and liposarcoma were the most common histological categories of STS in this study population.

Among patients initiating first-line systemic therapy, the most common regimen was docetaxel plus gemcitabine combination therapy (26.5%); the next most common first-line regimen (18.8%) was doxorubicin only. Both a recent medical record review study in the US [25] and the Sarcoma Treatment and Burden of Illness in North America and Europe chart review study [26] found that anthracyclines were used most commonly among first-line therapies (either alone or in combination) in younger patient populations. In this study of older adults, docetaxel plus gemcitabine was the most commonly used first-line therapy for most histologic categories except for vascular sarcomas (for which paclitaxel was most commonly used) and liposarcoma (for which doxorubicin was most commonly used). The use of paclitaxel for vascular sarcomas is consistent with evidence suggesting that vascular sarcomas may be relatively more responsive to taxanes and as such are recommended by NCCN guidelines [24, 28–30]. A relatively lower use of doxorubicin or related drugs was observed in this study, plausibly because the older age of the study population (mean age, 77.8 years) may increase concerns about adverse events, particularly doxorubicin-associated cardiotoxicity [27]. In the current study, 18.3% of the patients had a history of congestive heart failure and 25.8% of patients had a history of chronic pulmonary disease. Additionally, 69.2% of the patients in this study had hypertension that, along with age, is known to increase the risk of doxorubicin-associated cardiotoxicity [31, 32]. Prior research has addressed the issues of doxorubicin-based therapies and cardiovascular risk factors in the older adult population [33–35]. It is possible that the low use of doxorubicin observed in this study cohort is related to these issues, but the study design and available data do not allow for a thorough investigation of this potential relationship. Further understanding of physician preferences with regard to administration of doxorubicin-based therapy among older adult patients is needed to adequately comprehend observed treatment patterns. Knowledge about the appropriate care of the older patient remains limited, but data from this study begin to fill this gap and suggest directions for future research related to toxicity risk, disease progression, recurrence and survival outcomes, and their relationship with patient age in the setting of advanced STS.

As observed in other studies, gemcitabine was used most commonly during second-line therapy (either alone or in combination) [25, 26]. We also noted that dexrazoxane was not at all commonly used in this population among those who were treated with doxorubicin.

Approximately 62% of the study population received cancer-directed therapy. As compared to patients receiving cancer-directed therapy, patients receiving supportive care only had a mean age of 80.0 years (cancer-directed therapy: 76.5 years) at the time of being diagnosed with advanced disease and had a baseline comorbidity burden (mean CCI score) of 3.1 (cancer-directed therapy: 2.5), and 24.5% (cancer-directed therapy: 14.6%) had a history of congestive heart failure. Therefore, it seems likely that many of these patients may have been considered to not be candidates for chemotherapy, particularly with a cardiotoxic agent. The mean non-cancer CCI scores observed in this study for the overall population, patients receiving cancer-directed treatment, and patients receiving supportive care only are in line with those reported by Davis et al. among metastatic lung cancer patients [36].

Median overall survival in the entire study population was estimated to be less than 9 months. Patients who received supportive care lived only less than 3 months. The shorter survival of the group that received only supportive care, in addition to being related to not receiving anticancer treatment per se, is likely to be confounded by selection of patients for treatment who had a more favorable prognosis or better ability to tolerate treatment.

A study by Italiano et al. [7] (median age range 53–59 years), which included patients from the time frame of this study (i.e., 2002–2006), found that patients with synchronous or metachronous metastatic STS had an overall survival of 18 months from the time of metastatic diagnosis. Patients in the study by Italiano et al. [7] had relatively longer survival than those in this study, plausibly because patients in this study were considerably older. Similar to this study, survival did vary by histologic category in the study by Italiano et al. [7]. For example, patients with leiomyosarcoma, UPS, and nerve sheath sarcoma had a median survival of 12.9, 9.6, and 6.2 months, respectively, in this study and 19.4, 11.2, and 8.6 months, respectively, in the Italiano et al. [7] study.

An array of treatments (e.g. eribulin, olaratumab, pazopanib, trabectedin) became available for this population in the last decade [11, 13, 14, 37]; however, effectiveness of these treatments specifically among older adult patients with advanced STS is yet to established. Other treatments like oral cyclophosphamide plus prednisone [34] may also be feasible for older adult patients for whom treatment with doxorubicin may not be an option. Overall survival, progression-free survival, and response rates of patients with advanced STS may improve as these treatments will become part of routine care provided to this population.

This study is subject to several limitations inherent in analyses of Medicare claims data and the use of such data in studies of advanced cancer, in particular. For patients who were not initially diagnosed at the metastatic stage of the disease, ICD-9-CM diagnosis codes were used to identify evidence of metastatic disease during the follow-up period. However, the use of ICD-9-CM codes to identify metastatic disease has been shown to have sensitivity, specificity, and positive predictive value of less than 80%, and thus the use of ICD-9-CM diagnosis codes in the Medicare claims data may have resulted in inaccurate or under-identification of an advanced STS population [38, 39]. As described in the study methods, one of the criteria used to identify progression to metastatic disease was the initiation of systemic therapy at least 6 months after surgery. This criterion may have resulted in selection bias because patients who had disease progression but did not receive systemic therapy would have been omitted from the supportive care-only group. Although, the 6-month lag period after surgery was used to avoid interpreting adjuvant therapy as treatment for advanced disease, patients who progressed and received systemic therapy for advanced disease would have been omitted from the cancer-directed therapy group. Lines of therapy are not reported in claims data; therefore, an algorithm had to be defined to estimate the lines of therapy. This may have misclassified treatments by line of therapy, as the reasons for treatment changes were not available in the data. The Medicare Part D database was available for only a subset of the cohort (32.4%); however, only a small proportion of all chemotherapy (2%) claims were identified from the Medicare Part D database, so the risk of missing important treatment data is relatively low despite this limitation. An array of treatments (i.e., eribulin, olaratumab, pazopanib, trabectedin) became available for this population in the last decade, although these agents are not fully represented in the study dataset. Future research should evaluate the use and outcomes of these novel treatments in the older adult population. Finally, this study included only patients aged 65 years or older, and although SEER-Medicare data is representative of the US population 65 years and older for age and gender, participating SEER sites may not be representative with regard to distribution of race, income, urban residence, HMO enrollment as well as cancer mortality [15] and thus the results should not be generalized to the entire population of older adult patients with advanced STS.

Despite these limitations, this study documents real-world treatment patterns that may help inform providers, researchers, and policymakers about the care of older patients with STS in the US. As real-world data including new therapeutic options become available, our results provide a basis for analyzing changes in treatment patterns and outcomes over time. This study demonstrates that the prognosis is poor for older adult patients with advanced STS, highlighting the unmet medical need in this population.

## Conclusion

Although previous studies of younger populations reported anthracycline-based therapy predominated in first line, our study of older adults with advanced STS found that doxorubicin was not commonly used. Despite variation by histologic category, prognosis was observed to be poor for older adult patients with advanced STS in this study.

### Additional file

**Additional file 1: Table S1.** Patient Characteristics by Treatment Status (Received Cancer-Directed Treatment or Supportive Care Only).

## Appendix 1

See Table 6.

**Table 6:** Advanced soft-tissue sarcoma histologic categories and subtypes. Source: World Health Organization [17]

Histologic subtype (ICD-O-3)	Description^a^
Fibroblastic/myofibroblastic sarcomas	
8810/3	Fibrosarcoma, NOS
8811/3	Fibromyxosarcoma
8814/3	Infantile fibrosarcoma
8815/3	Solitary fibrous tumor, malignant
8825/3	Myofibroblastic sarcoma
8832/3	Dermatofibrosarcoma, NOS
Leiomyosarcomas	
8890/3	Leiomyosarcoma, NOS
8891/3	Epithelioid leiomyosarcoma
8896/3	Myxoid leiomyosarcoma
Liposarcomas	
8850/3	Liposarcoma, NOS
8851/3	Liposarcoma, well differentiated
8852/3	Myxoid liposarcoma
8853/3	Round cell liposarcoma
8854/3	Pleomorphic liposarcoma
8855/3	Mixed liposarcoma
8857/3	Fibroblastic liposarcoma
8858/3	Dedifferentiated liposarcoma
Nerve sheath sarcomas	
9540/3	Malignant peripheral nerve sheath tumor
9560/3	Malignant neurilemoma
9561/3	Malignant triton tumor
9571/3	Malignant Perineurioma
9580/3	Malignant granular cell tumor
Others/NOS	
8800/3	Sarcoma, NOS
8801/3	Undifferentiated spindle cell sarcoma
8802/3	Undifferentiated pleomorphic sarcoma
8803/3	Undifferentiated round cell sarcoma
8804/3	Undifferentiated epithelioid sarcoma
8805/3	Undifferentiated sarcoma
8806/3	Desmoplastic small round cell tumor
8840/3	Myxosarcoma
8842/3	Ossifying fibromyxoid tumor, atypical
8860/3	Angiomyolipoma
8894/3	Angiomyosarcoma
8895/3	Myosarcoma
8940/3	Mixed tumor, malignant, NOS
8963/3	Malignant rhabdoid tumor
8982/3	Myoepithelial carcinoma
8990/3	Mesenchymoma, malignant
9020/3	Phyllodes tumor, malignant
9044/3	Clear cell sarcoma, NOS (except of kidney)
9133/3	Epithelioid hemangioendothelioma, malignant
9231/3	Myxoid chondrosarcoma
9251/3	Malignant giant cell tumor of soft parts
9364/3	Peripheral neuroectodermal tumor
Rhabdomyosarcomas	
8900/3	Rhabdomyosarcoma, NOS
8901/3	Pleomorphic rhabdomyosarcoma, adult type
8902/3	Mixed type rhabdomyosarcoma
8910/3	Embryonal rhabdomyosarcoma, NOS
8912/3	Spindle cell rhabdomyosarcoma
8920/3	Alveolar rhabdomyosarcoma
Synovial sarcomas	
9040/3	Synovial sarcoma, NOS
9041/3	Synovial sarcoma, spindle cell
9042/3	Synovial sarcoma, epithelioid cell
9043/3	Synovial sarcoma, biphasic
Undifferentiated pleomorphic sarcoma (previously known as malignant fibrous histiocytoma or MFH)	
8830/3	Malignant fibrous histiocytoma
Vascular sarcomas	
9120/3	Hemangiosarcoma
9130/3	Hemangioendothelioma, malignant
9150/3	Hemangiopericytoma, malignant
9161/3	Hemangioblastoma; angioblastoma
9170/3	Lymphangiosarcoma

*NOS* not otherwise specified

## Appendix 2

See Table 7.

**Table 7:** Patient and tumor characteristics, overall and by histologic categories

	All patients	Leiomyo-sarcomas	Undifferentiated pleomorphic sarcoma	Liposarcomas	Vascular sarcomas	Fibroblastic/myofibroblastic sarcomas	Nerve sheath sarcomas	Rhabdomyo-sarcomas	Synovial sarcomas	Others/NOS
N	4274	922	652	554	357	227	106	98	49	1309
Location of residence (n, %)										
Big metro	2358 (55.2)	507 (55.0)	362 (55.5)	294 (53.1)	198 (55.5)	133 (58.6)	55 (51.9)	54 (55.1)	25 (51.0)	730 (55.8)
Less urban	1269 (29.7)	267 (29.0)	187 (28.7)	172 (31.1)	111 (31.1)	65 (28.6)	40 (37.7)	29 (29.6)	16 (32.7)	382 (29.2)
Metro	255 (6.0)	55 (6.0)	39 (6.0)	35 (6.3)	21 (5.9)	–	–	–	–	83 (6.3)
Rural	320 (7.5)	78 (8.5)	60 (9.2)	41 (7.4)	–	17 (7.5)	–	–	–	87 (6.7)
Urban	71 (1.7)	15 (1.6)	–	12 (2.2)	–	–	0 (0.0)	–	0 (0.0)	27 (2.1)
SEER region (n, %)										
Northeast	981 (23.0)	197 (21.4)	147 (22.6)	143 (25.8)	75 (21.0)	59 (26.0)	29 (27.4)	24 (24.5)	13 (26.5)	294 (22.5)
Midwest	472 (11.0)	124 (13.5)	57 (8.7)	47 (8.5)	35 (9.8)	29 (12.8)	12 (11.3)	13 (13.3)	–	149 (11.4)
South	971 (22.7)	238 (25.8)	170 (26.1)	106 (19.1)	94 (26.3)	27 (11.9)	16 (15.1)	18 (18.4)	–	292 (22.3)
West	1850 (43.3)	363 (39.4)	278 (42.6)	258 (46.6)	153 (42.9)	112 (49.3)	49 (46.2)	43 (43.9)	20 (40.8)	574 (43.9)
Anatomic location of primary tumor at the time of initial diagnosis of STS (n, %)										
Axilla	526 (12.3)	110 (11.9)	65 (10.0)	105 (19.0)	41 (11.5)	37 (16.3)	13 (12.3)	–	–	140 (10.7)
Breast	123 (2.9)	12 (1.3)	–	–	28 (7.8)	–	–	–	0 (0.0)	67 (5.1)
Genitourinary	106 (2.5)	34 (3.7)	–	–	12 (3.4)	–	–	–	–	38 (2.9)
Head or neck	354 (8.3)	31 (3.4)	104 (16.0)	13 (2.4)	108 (30.3)	13 (5.7)	17 (16.0)	–	–	61 (4.7)
Lower limb	937 (21.9)	125 (13.6)	236 (36.2)	122 (22.0)	41 (11.5)	58 (25.6)	22 (20.8)	19 (19.4)	16 (32.7)	298 (22.8)
Mediastinum, lung, or pleura	221 (5.2)	29 (3.2)	12 (1.8)	–	16 (4.5)	28 (12.3)	–	–	–	110 (8.4)
Pelvis	416 (9.7)	83 (9.0)	66 (10.1)	49 (8.8)	30 (8.4)	23 (10.1)	11 (10.4)	–	–	144 (11.0)
Retroperitoneal	396 (9.3)	118 (12.8)	19 (2.9)	174 (31.4)	–	–	–	–	0 (0.0)	70 (5.4)
Trunk	283 (6.6)	59 (6.4)	39 (6.0)	26 (4.7)	25 (7.0)	16 (7.1)	–	–	–	99 (7.6)
Upper limb	309 (7.2)	36 (3.9)	89 (13.7)	32 (5.8)	11 (3.1)	30 (13.2)	–	–	–	87 (6.7)
Uterus	366 (8.6)	228 (24.7)	–	15 (2.7)	0 (0.0)	–	–	21 (21.4)	0 (0.0)	92 (7.0)
Other	237 (5.6)	57 (6.2)	–	–	41 (11.5)	–	–	–	–	103 (7.9)

## Abbreviations

CCI: Charlson Comorbidity Index
CI: confidence interval
HCPCS: Healthcare Common Procedure Coding System
ICD-9-CM: International Classification of Diseases, 9th Revision, Clinical Modification
ICD-O-3: International Classification of Diseases for Oncology, 3rd Edition
NCCN: National Comprehensive Cancer Network
NCI: National Cancer Institute
NOS: not otherwise specified
SD: standard deviation
SEER: The Surveillance, Epidemiology, and End Results
STS: soft-tissue sarcoma
UPS: undifferentiated pleomorphic sarcoma
US: United States
