# Supplementary material for: Treatment patterns and survival among older adults in the United States with advanced soft-tissue sarcomas
Source: Clin Sarcoma Res. 2018 May 3;8:8. doi: 10.1186/s13569-018-0094-x (PMC5932822; doi:10.1186/s13569-018-0094-x)
Supplement: Supplementary file 1 — Additional file 1: Table S1. Patient Characteristics by Treatment Status (Received Cancer-Directed Treatment or Supportive Care Only). [file 13569_2018_94_MOESM1_ESM.docx]

**Table S1. Patient Characteristics by Treatment Status (Received Cancer-Directed Treatment or Supportive Care Only)**

|  | **Received Cancer-Directed Treatment** | | **Received Supportive Care Only** | |
| --- | --- | --- | --- | --- |
| n, % | 2,656 | 62.1% | 1,618 | 37.9% |
| Sex (n, %) |  |  |  |  |
| Male | 1,333 | 50.2% | 770 | 47.6% |
| Female | 1,323 | 49.8% | 848 | 52.4% |
| Race |  |  |  |  |
| White | 2,350 | 88.5% | 1,379 | 85.2% |
| Black | 191 | 7.2% | 146 | 9.0% |
| Other | 111 | 4.2% | 86 | 5.3% |
| Unknown | 0 | 0.0% | 0 | 0.0% |
| Age at *advanced* diagnosis |  |  |  |  |
| Mean (SD) | 76.5 | 6.9 | 80.0 | 7.5 |
| Median | 75.9 |  | 80.0 |  |
| Range (minimum, maximum) | 65.0 | 97.1 | 65.1 | 104.1 |
| Distribution (n, %) |  |  |  |  |
| 65-69 | 563 | 21.2% | 173 | 10.7% |
| 70-74 | 624 | 23.5% | 287 | 17.7% |
| 75-79 | 641 | 24.1% | 348 | 21.5% |
| 80-84 | 477 | 18.0% | 381 | 23.6% |
| 85+ | 351 | 13.2% | 429 | 26.5% |
| Pre–index date CCI score^a^ |  |  |  |  |
| Mean (SD) | 2.5 | 2.1 | 3.1 | 2.6 |
| Median | 2.0 |  | 2.5 |  |
| Minimum, maximum | 0.0 | 14.0 | 0.0 | 15.0 |
| Distribution (n, %) |  |  |  |  |
| 0 | 377 | 14.2% | 197 | 12.2% |
| 1 | 622 | 23.4% | 293 | 18.1% |
| 2 | 550 | 20.7% | 319 | 19.7% |
| 3 | 422 | 15.9% | 223 | 13.8% |
| 4 | 255 | 9.6% | 184 | 11.4% |
| 5+ | 430 | 16.2% | 402 | 24.9% |
| CCI Conditions During Pre-Index Date Period (n, %) |  |  |  |  |
| Cerebrovascular disease | 303 | 11.4% | 275 | 17.0% |
| Chronic pulmonary disease | 648 | 24.4% | 454 | 28.1% |
| Congestive heart failure | 388 | 14.6% | 396 | 24.5% |
| Dementia | 51 | 1.9% | 104 | 6.4% |
| Depression | 184 | 6.9% | 157 | 9.7% |
| Diabetes w/complications | 174 | 6.6% | 119 | 7.4% |
| Diabetes without complications | 755 | 28.4% | 465 | 28.7% |
| Hemiplegia or paraplegia | 26 | 1.0% | 36 | 2.2% |
| HIV/AIDS | 0 | 0.0% | – | – |
| Hypertension | 1831 | 68.9% | 1126 | 69.6% |
| Mild liver disease | 139 | 5.2% | 104 | 6.4% |
| Moderate-to-severe liver disease | – | – | – | – |
| Myocardial infarction | 161 | 6.1% | 117 | 7.2% |
| Peptic ulcer disease | 64 | 2.4% | 46 | 2.8% |
| Peripheral vascular disease | 481 | 18.1% | 366 | 22.6% |
| Renal disease | 216 | 8.1% | 203 | 12.6% |
| Rheumatic disease | 116 | 4.4% | 58 | 3.6% |
| Skin ulcers/cellulitis | 346 | 13.0% | 283 | 17.5% |
| Takes warfarin | 84 | 3.2% | 53 | 3.3% |
| Stage of disease at initial diagnosis of STS (n, %) |  |  |  |  |
| Localized | 1060 | 39.9% | 671 | 41.5% |
| Regional - direct extension only | 614 | 23.1% | 324 | 20.0% |
| Regional - lymph nodes involved only | 24 | 0.9% | – | – |
| Regional - direct extension and lymph nodes | 23 | 0.9% | – | – |
| Distant | 935 | 35.2% | 604 | 37.3% |

AIDS, acquired immune deficiency syndrome; CCI, Charlson Comorbidity Index; HIV, human immunodeficiency virus; ICD-9-CM, International Classification of Diseases, 9th Revision, Clinical Modification; SD, standard deviation; STS, soft tissue sarcoma.

^a^ Because the objective of the CCI score was to evaluate underlying comorbidity burden independent of STS, ICD-9-CM diagnosis codes for cancer were excluded from the CCI calculation for this study.
